# Crossword expertise as recognitional decision making: an artificial intelligence approach

**DOI:** 10.3389/fpsyg.2014.01018

**Published:** 2014-09-11

**Authors:** Kejkaew Thanasuan, Shane T. Mueller

**Affiliations:** Department of Cognitive and Learning Sciences, Michigan Technological UniversityHoughton, MI, USA

**Keywords:** crossword puzzles, recognitional decision making, AI, expertise, lexical memory search

## Abstract

The skills required to solve crossword puzzles involve two important aspects of lexical memory: semantic information in the form of clues that indicate the meaning of the answer, and orthographic patterns that constrain the possibilities but may also provide hints to possible answers. Mueller and Thanasuan (2013) proposed a model accounting for the simple memory access processes involved in solving individual crossword clues, but expert solvers also bring additional skills and strategies to bear on solving complete puzzles. In this paper, we developed an computational model of crossword solving that incorporates strategic and other factors, and is capable of solving crossword puzzles in a human-like fashion, in order to understand the complete set of skills needed to solve a crossword puzzle. We compare our models to human expert and novice solvers to investigate how different strategic and structural factors in crossword play impact overall performance. Results reveal that expert crossword solving relies heavily on fluent semantic memory search and retrieval, which appear to allow experts to take better advantage of orthographic-route solutions, and experts employ strategies that enable them to use orthographic information. Furthermore, other processes central to traditional AI models (error correction and backtracking) appear to be of less importance for human players.

## 1. Introduction

Crossword puzzles were first introduced in 1913, and have become both a popular pastime, mental training aid, and a domain of study for psychological researchers (e.g., Nickerson, 2011), who have long acknowledged the role of memory access in puzzle solving. Previously, Mueller and Thanasuan (2014) we proposed a model of the basic memory search processes involved in solving individual crossword clues, and suggest that the joint access and constraint provided by cues in crossword puzzles make it similar to expert decision making in many domains.

For many of the same reasons that make them engaging puzzles for humans, crossword puzzles also pose an interesting problem for Artificial Intelligence (AI) systems, as solving them requires using many of the fundamental aspects of modern AI: search, heuristics, constraint satisfaction, knowledge representation, optimization, and data mining. Because crossword solving requires searching simultaneously within two distinct spaces (i.e., semantic and orthographic), and easily permits backtracking and recursion, it is also a useful problem for learning and teaching AI (e.g., Ginsberg et al., 1990; Harris et al., 1993; Shazeer et al., 1999; Littman et al., 2002). “Dr. Fill” (Ginsberg, 2011) is currently the best-known and most advanced AI crossword solver, and it typically performs perfectly on nearly all “straight” puzzles, only making mistakes on puzzles with complex or unusual themes or letter arrangements (Lohr, 2012). For example, when competing at the 2012 American Crossword Puzzle Tournament (ACPT), Dr. Fill failed on a puzzle in which many of the answers were required to be filled in backward, a twist that also challenged many human solvers. Dr. Fill finished the 2012 ACPT 141st of approximately 600 contestants and improved to 92nd place in 2013, and 67th place in 2014. The improvement over time is related not only to broader knowledge corpora being used, but also the incorporation of more rules for handling tricky puzzle themes, which often include puns, rebuses (i.e., letter substitutions), and other wordplay devices.

Although Dr. Fill illustrates that AI can be competitive with the best human players, AI systems typically use very non-human strategies to accomplish this. In arriving at a final answer, they may end up solving a puzzle dozens or hundreds of times, selecting the solution that best fits many constraints. In contrast, human solvers use a different combination of skills, including decision making, pattern recognition (Grady, 2010), lexical memory access (Nickerson, 1977) and motor skills such as typing or moving in a grid. Speed-solvers develop these skills to challenge themselves, to enable solving more puzzles per day (often five or six), and to compete in competitions. They tend not to use backtracking or error correction extensively (at least to the extent that computerized systems do), and they are minimally impacted by difficulty (see Mueller and Thanasuan, 2013). Moreover, they still outperform AI solutions on puzzles that are moderately challenging.

Although AI crossword solvers can complete many puzzles almost perfectly, these systems tend not to be based on human strategies or known human memory structure. In this paper, we adopt a Biologically-Inspired Artificial Intelligence approach (see Samsonovich and Mueller, 2008) to understand human expert crossword play, derived from assumptions about the lexical access routes and solution strategies of expert crossword players. We will use this model to understand the relative contributions of different types of knowledge and strategies to crossword play, in an effort to understand some of the cognitive skills that are highly developed in superior crossword players.

## 2. An recognition-primed AI model of crossword play

In many domains, expert decisions appear to be described by the Recognition-Primed Decision (RPD) model (Klein, 1993). Although many decision theories focus on making choices between clearly-defined options that often embody trade-offs, RPD argues that what makes experts good at what they do is in their ability to quickly generate and evaluate a single workable candidate solution from their vast knowledge and experience (rather than weighing and comparing options). For example, Klein et al. (1986) applied the model to a fireground incidents and found that, rather than selecting between courses of action, fireground commanders typically selected the first option that came to mind and adapted it to fit the current situation (akin to the “take the first” strategy hypothesized by Johnson and Raab, 2003). This maps onto the phenomenology of crossword play–rarely are players choosing between options to determine which is best^1^. Instead, solvers either know the answer, or do not. In contrast to the types of situations to which RPD has typically been applied, crossword play does not permit approximate solutions, and so the decision problem is one where a player must determine whether or not they know the exact answer, and if they do not know the answer, they must decide how to continue search (i.e., either via continued memory search, generating more candidates through associative memory, or by trying to obtain more letter hints by solving other clues).

**Figure 1:**
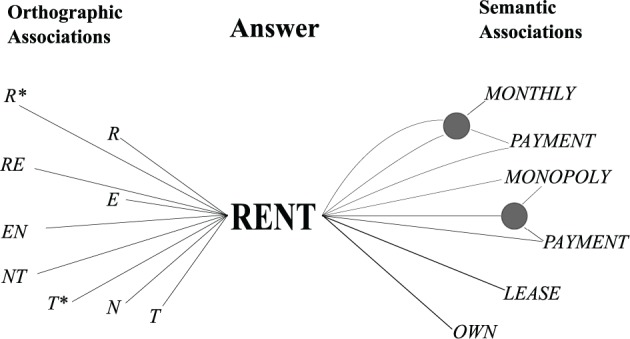
**The example of semantic and orthographic routes**.

Mueller and Thanasuan (2013) described and developed a crossword solving model by modifying the Bayesian Recognitional Decision Model (BRDM; Mueller, 2009), a Bayesian implementation of the RPD model. The model implements a decision process via memory retrieval, and the basic mechanisms originate from models of recognition memory (Raaijmakers and Shiffrin, 1981), although the basic notion of experience-based and case-based decision making has been explored in a number of computational models (Dougherty et al., 1999; Warwick et al., 2001; Sokolowski, 2003; Ji et al., 2007; Thomas et al., 2008). The basic procedure applies two independent routes to solve a crossword clue:

- A semantic route: the model takes clue-word associations as cues to search for possible answers and checks them with an orthographic cue for feasibility. An example of clue-word associations is shown in Figure 1.
- An orthographic route: the model uses letter combinations and letter-word associations to generate candidate answers. The example is shown in Figure 1. These candidates are checked for semantic similarity and pattern matches.

Both routes adopt the same basic retrieval mechanism based on previous models of recognitional decision making. This mechanism was explored in its simplest form in Mueller and Thanasuan (2014) as a model of word-stem completion, and more fully in Mueller and Thanasuan (2013) for both orthographic and semantic routes. We will first describe the basic memory retrieval mechanisms. The form we use simplifies the Bayesian calculation in the BRDM model proposed by (Mueller, 2009) (which makes some of the computations easier on the large corpus), but in practice the rank-order distributions produced by the present model are nearly identical to those produced by the BRDM implementation.

### 2.1. Retrieval mechanisms

Our approach to modeling crossword play is grounded in memory retrieval described by Mueller and Thanasuan (2013), with the addition of a memory processing time parameter so that we can make predictions about performance time. We hypothesize that both the orthographic and semantic routes work similarly, but since their information is from different modalities, they cannot be combined in order to simultaneously probe memory. Although this assumption differs from the conclusions reached by Massaro et al. (1991) on a similar task (discussed in Mueller and Thanasuan, 2013), our two-routes hypothesis is simpler, is sufficient to model our data, and alternatives produce results that are generally difficult to distinguish from the version we use here. Instead, each route is probed independently, the two candidate answers are evaluated with respect to their association strength to the clue, and the alternative with greater strength is used. We assume that the strength between a word and its associations (either word parts or clue parts) is learned via a simple model based on Estes (1950) stimulus sampling theory. As more and more associations are learned, the strength between each word and its associates grows and asymptotes to a finite level, but even though a single word-word association may be strong, each associate competes with other associates, making specific associations difficult to access.

Once learned, a set of features present in a clue will have strengths of association to many possible answers. We assume the search and identification process is both a logical and a probabilistic process. First, a set of association strengths is computed between any cue hint (e.g., a letter, letter pair, word, or word bigram) over all possible answers, for either the orthographic (*Pr^O^*) or semantic (*Pr^S^*) memory:

(1) $$Pr^{O}(A_{i}|u_{j})\;=\;O_{ij}/\sum_{i}O_{ij}$$

(2) $$Pr^{S}(A_{i}|u_{j})\;=\;S_{ij}/\sum_{i}S_{ij}$$

where *u_j_* represents either semantic or orthographic features indexed by *j* and *A_i_* is a candidate answer *i*. Thus, the strength of association between any feature and any cue is monotonically related to the frequency with which that cue tends to be have appeared with that answer.

Any clue *u* will consist of a set of features *u_j_*, and we compute the joint probability of that set via the *n^th^* root of the product of each individual probability (plus a smoothing constant σ). Here, *n* refers to the number of features in *u*. First, the strength *B* for each clue is computed, after which it is normalized based on the strength for all possible clues to compute *Pr^X^*(*A_i_*|*u*) (substituting *X* for *O* or *S*):

(3) $$B(A_{i}|u)\;=\;{\left(\prod_{j\;\in \;u}Pr^{X}(A_{i}|u_{j})+\sigma \right)}^{\left(1/n\right)}$$

(4) $$Pr^{X}(A_{i}|u)\;=\;B(A_{i}|u)/\sum_{i}B(A_{i}|u)$$

The probability value *Pr^X^*(*A_i_*|*u*) provides a strength index indicating the relative likelihood of different candidate answers coming to mind, given a particular clue.

### 2.2. Search, recovery, and checking mechanisms

This basic memory retrieval mechanism described above will lead to a rank-order set of activations that produce candidate solutions activated by either orthographic or semantic information. Much like previous models of memory retrieval (Raaijmakers and Shiffrin, 1981), we assume that this provides an activation distribution that enables memory “images” to be identified. For orthographic cues, the retrieval results in a complete word that tends to contain the features in the cue. For semantic cues, we assume the retrieval identifies an concept whose specific lexical form still needs to be recovered. The probability of recovery is determined by *Pr^S^*(*A_i_*|*u*) in Equation (4), and a recovery or fluency parameter whose value we assume may differ as a function of expertise:

(5) $$Pr_{recovery}\;=\;1\;-\;exp\left(-Pr^{S}(A_{i}|u)\;*\;recovery\right)$$

Consequently, Equation (5) provides one potential source for modeling expertise. Our assumption is that experts may be especially fluent at recovering lexical exemplars associated with a concept, even if the answer could be recognized as correct if provided. Although this is most easily interpreted as the probability of generating the “surface features” of particular word based on a semantic gist “image,” it could also represent other more conceptual memory retrieval failures that also differ between more traditional memory paradigms such as recognition memory and free recall^2^. By using the recovery probability to model expertise, it represents several related aspects of fluency, but it remains an open question of whether crossword experts are especially fluent for both surface features and deeper semantic or episodic associations.

In our models, the recovery parameter also stands in for the overall richness of the knowledge base. Our expert and novice models both use the same knowledge-base corpus. Although experts clearly have a richer body of crossword-specific knowledge, and likely have broader general knowledge (cf. Hambrick et al., 1999), we have elected to not use separate corpora for experts and novices, for several reasons. First, our experience is that the answers to most clues are recognizeable by most people once the answer is revealed. This indicates that most of the knowledge in a crossword is available in latent form that can be recognized but not retrieved, which maps closely onto our recovery parameter. A second concern is that the clue data we employ is large enough that we found it impractical to create multiple versions for experts and novices, and so a using the recovery parameter is a simple way to make part of the expert lexicon inaccessible to novices.

### 2.3. Timing

Retrieval time for declarative information has long been assumed to be related to activation strength of the facts being recalled (see Lewis and Vasishth, 2005). Although it is difficult to predict how changes in the lexicon will impact timing (as it may lead to a greater competition for activation), it is certainly true that experts must retrieve facts very quickly in order to solve the puzzle. However, several aspects of timing are involved in solving a clue, which we can separate into four operators: moving, reading, typing, and retrieving.

(6) $$T_{solving}\;=\;d\;*\;t_{moving}\;+\;t_{reading}\;+\;n\;*\;t_{retrieval}\;+\;wl\;*\;t_{typing}$$

where *t_reading_* represents the time that participant spends reading a clue, *n* is the number of candidate answers that the model generates before it gets the first one that fit the orthographic pattern, *t_retrieval_* is the generating and checking time for each candidate answer. *wl* is a word length and *t_typing_* is the average typing time, *t_moving_* is the time required to move between adjacent cells, while *d* is the number of moves needed to go to the first letter cell of the next clue (i.e., the Manhattan distance).

Although any of these may differ between novices and experts, it can be difficult to separate these in a naturalistic context. Consquently, we will use default values (estimated by Kieras, 2001) of 0.28 s for the typing time, 0.14 s for moving time, and 1.0 s for reading time, for all users. Thus, we have elected to attributed all expert-novice differences to retrieval time. This assumption is probably incorrect, because experts have a lot of experience navigating in crossword software, and are typically intrinsically motivated to be fast.

In general, *t_retrieval_* could be computed based on memory activation directly, using for example the ACT-R retrieval time equation (*RT* = *Fe^fA^_i_*). In the present simulations, we will allow retrieval time to vary independently, to investigate how speed on its own might explain expert-novice differences.

### 2.4. Gridfill strategy

Another way in which experts may differ from novices is via the strategy by which they choose the next clue to solve. For example, experts appear to be more likely to attempt clues that are already partially solved, as well as those close to the last solved clue, rather than picking easy clues far from a previously-solved clue^3^. Although strategies may differ between novices and experts, it is unclear whether they have a large or small impact on overall performance. If the main time bottleneck is memory retrieval, then changing gridfill strategies may only increase overall solution times marginally. This may help an expert reduce their solution time by a few seconds, but would not enable a novice to become an expert. To investigate this, we will examine whether gridfill strategy play a role in expertise.

The first strategy is one we refer to as a *Random movement strategy*. Using this strategy, players simply choose a random un-answered clue to attempt to solve next. This model provides a least-informed but reasonable strategy that may provide a lower bracket on performance. The second strategy, which we refer to as an *Optimizing movement strategy*, attempts to select clues that (1) are partially filled; (2) are close to the current clue; and (3) have not been attempted previously. As we will discuss, neither of the strategies uses extensive error detection, error correction, or backtracking, which is roughly consistent with observed crossword play.

### 2.5. Overall crossword solver

**Figure 2:**
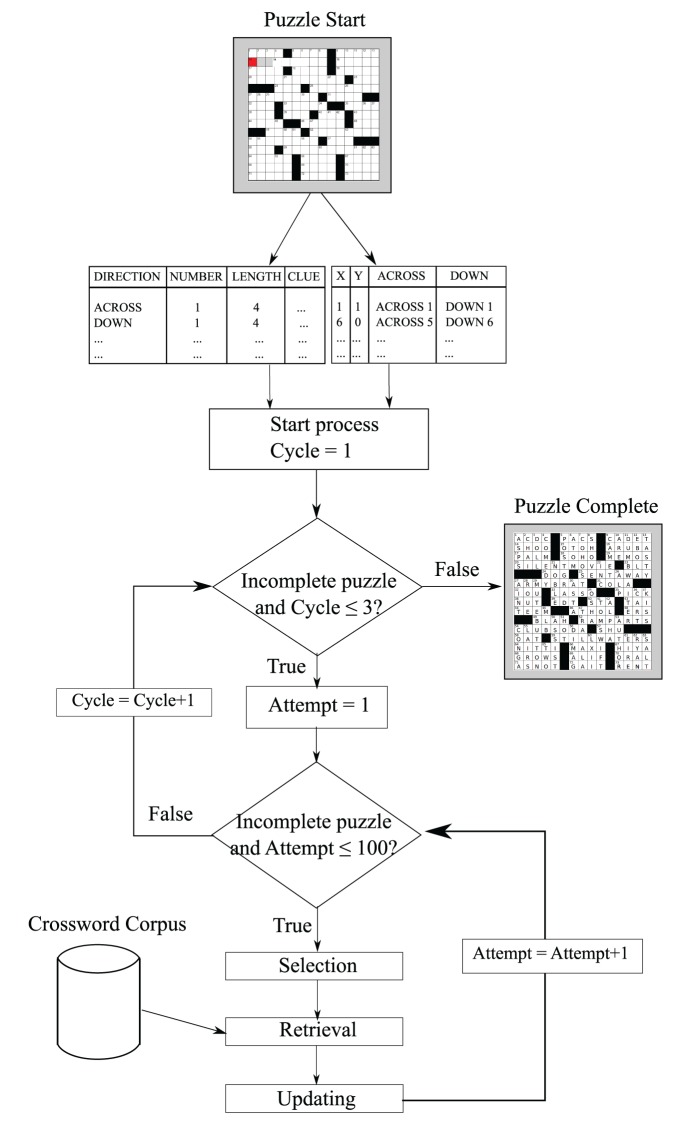
**The overall solution strategy.** The model iteratively selects clues, attempts to solve one, and then updates the current state of the grid.

The solver we ultimately created does not view the crossword grid visually, but rather has access to all clues and word patterns from the grid puzzle directly (see Figure 2) in the form of two tables. The first table depicts position coordinates corresponding to the clues. The second table contains essential variables such as word lengths, clues, directions, and start positions. The AI algorithm can be segmented into three stages: selection, retrieval, and updating. The selection process describes how we select a clue to solve based on the current state of the puzzle. For the retrieval process, if no orthographic information (other than word length) is present, only semantic cues are used. Otherwise, both semantic and orthographic routes are employed independently to retrieve candidate answers. Each retrieval route process returns the first answer that fits the word pattern (consistent with Mueller and Thanasuan, 2013, which fit data only from individual clues). Then, the semantic probabilities (i.e., the activation strength) of those answers from both routes is compared and the larger one is used as the best answer. The final process is updating. If an answer is returned from the retrieval process, the crossword status is updated to reflect new filled letters and completed words, in both across and down orientations.

The optimizing solver must have some ability to determine when search for a clue has failed so that it should give up and move on to another clue, to avoid getting stuck repeatedly trying to solve the same “best” clue. Normally, the model selects (probabilistically) the best clue to attempt, but if it fails, it could end up oscillating between one or two “best” options that it repeatedly fails at. To deal with this, we implement a strategy to avoid revisiting failed clues, using counters shown in Figure 2 as **Cycle** and **Attempt** (that maps roughly onto a an activation marking past search; cf. Mueller et al., 2013). Although this particular implementation is somewhat ad hoc, the basic process is representative of a class of strategies that attempt to seek out novelty. The counter is incremented any time an attempt to solve a clue is made.

Although we believe that neither experts nor novices use backtracking and error detection frequently, it certainly can happen, and this point in the cycling process could be used to signal an error that could lead to correction. This could be used to isolate an error to a small set of clues that could then be re-evaluated, “erased,” and re-solved. We have not implemented such a process in our current model, because the ability to backtrack (a core AI principle) can potentially hide the weaknesses of a less capable solver if used extensively.

### 2.6. Knowledge base

For the current demonstration, we use the associative knowledge base described by Mueller and Thanasuan (2013), relying solely on Ginsberg's crossword constructor clue-answer pairs database (http://www.otsys.com/clue/), which contains more than 4 million clues. However, other associative knowledge bases could be used to provide additional semantic and orthographic cues relevant to solving crossword puzzles. For orthographic knowledge, a set of associations between words and word parts must be inferred, and for semantic knowledge, a set of associations between answers and potential clue words and clue word combinations. This could incorporate free association norms (Nelson et al., 2004), semantic spaces derived from co-occurrence statistics, n-grams, WordNet (Miller et al., 1990), thesaurus (Samsonovich, 2014), and other sources. We suspect that additional knowledge bases would broaden the knowledge, but might ultimately reduce the specificity of associative cues and produce worse overall solvers.

## 3. Experiment

In order to test the ability of the proposed model to account for data, and to understand the relative importance of our hypothesized parameters, we conducted an experiment involving novices and experts attempting to solve a crossword puzzle.

### 3.1. Participants

We recruited 21 participants both from the Michigan Technological University undergraduate subject pool, and 14 crossword experts from attendees of the 2012 American Crossword Puzzle Tournament (ACPT). The study was approved through the Michigan Technological University Human Subjects Institutional Review Board, and were conducted under U.S. Federal human subjects guidelines. All participants had to read and either signed or clicked to accept an informed consent statement. Then, they were tested via instrumented computer software, undergraduates in a laboratory setting, and experts on their own computers via a downloadable software package.

### 3.2. Procedure

#### 3.2.1. Demographic survey

The study began with a brief computerized survey implemented using PEBL survey generator (Mueller and Piper, 2014), which included a series of questions related to personal experience with crosswords and related word games. Most undergraduate participants reported rarely playing crossword puzzles previously, although some had experience with related word games such as Scrabble, Bananagrams, Words with Friends, or Boggle. On the other hand, crossword experts reported playing puzzles on average more than 3 h per week (213 ± 149 min), and had been playing crossword puzzles for 15.3 ± 14.7 years.

#### 3.2.2. Crossword puzzle

**Figure 3:**
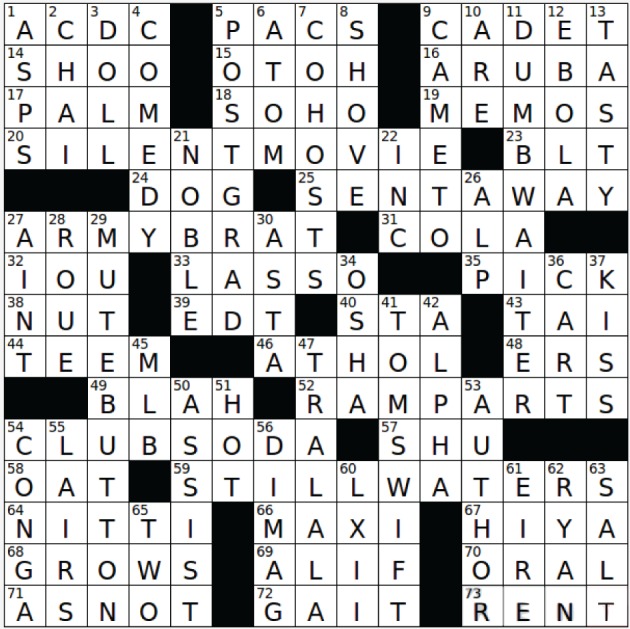
**The test puzzle used in the present experiment (“Quiet, Please” by Paula Gamache).** Used by permission.

Following the survey, participants solved two crossword puzzles using specially-developed software. We adapted the open source python-based application called XWord (http://sourceforge.net/projects/x-word/), which we instrumented to allow better control over data collection, and to improve data logging and keystroke-level recording. The software was adapted so that each clue was only viewable when the corresponding grid entry was selected, to enable us to better know how much time was spent looking at each clue. The first puzzle was a 4-min practice puzzle that allowed participants to become familiar with the control of the software. This was a standard sized puzzle (15 x 15), but along with each clue, the correct answer was provided. Most participants finished this puzzle in the allotted time. The second puzzle was a 78-clue 15 x 15 test puzzle, originally entitled “Quiet, Please” (Gamache, 2009), but with many of the clues edited to make them somewhat easier. Filled-in answers are shown in Figure 3, and the clues are shown in **Table 3**. The participants were instructed to solve the puzzle as fast as they could in 25 min. In addition to the puzzle and the survey, participants also took part in a stem completion test whose results are reported in Mueller and Thanasuan (2014).

## 4. Results

Results showed that the experts performed much better than novices in both speed and accuracy. No novice players could finish the puzzle in the 25 min allotted (average complete answers 23.9 ± 9.4 sd; correct answers = 23.1 ± 9.5). On the other hand, all expert players completed the puzzle (in 5.3 ± 2.5 min), with on average 77.2 out of 78 answers correct (± 1.5).

To examine performance differences between these two groups, we first inferred the cumulative time spent on each clue. Cumulative clue time is difficult to determine unambiguously in a natural crossword-solving setting, because a solver may revisit a word multiple times before an answer is completed, and some experts even “save up” an answer they have solved, entering it letter-by-letter when each cross-answer is solved. In addition, as the grid fills, the last letter of some clues will necessarily be filled while completing a crossing clue. Using several heuristics, we estimated cumulative response time for each clue by combining every time interval participants spent on each clue before they finished it. We then conducted a linear regression on log(cumulative response time) using answer length and the test clue frequencies (as they appear in the Ginsberg database) as predictors, along with a categorical predictor allowing the intercept to differ for each participant.

Within the crossword puzzles, shorter answers are more common, and this was true for the crossword we tested (ln(frequency in the lexicon) and word length were correlated with Pearson's R of −0.797). Because of this colinearity, it can be difficult to identify the source of length or frequency effects. A mixed-effects model (using the lmer function of the lme4 package of the R statistical computing language) treating participant and answer as random factors found the best-fitting model predicting ln(completion time) was −0.089 × *ln*(*freq* + 1) + 0.065*wl with distinct intercepts for experts (1.31) and novices (2.72). Separate χ^2^ tests comparing models with and without each predictor showed that the effects were each significant (for word length, χ^2^ = 6.26, *p* = 0.01, partial η^2^ = 0.009; for frequency, χ^2^ = 55,*p* < 0.001, partial η^2^ = 0.044, for expert status, χ^2^ = 56,*p* < 0.001, partial η^2^ = 0.58. Models that included expertise by word length or frequency interactions did not significantly improve the overall fit of the model, suggesting that as a first approximation, time factors that are related to length (such as typing time) does not differ between experts and novices.

As discussed in the description of the model, if we assume the time differences stem from cognitive processes (rather than motor processes) and use the average typing speed of 0.28 s per keystroke described by (Kieras, 2001), we can estimate memory retrieval times for the two groups. We do so by assuming the average solving time for each clue is the difference between cumulative response time for each clue and the sum of typing time of that clue. When adjusting in this way for word length, we found that the experts came up with an answer approximately six times faster than the novices did (novice: 17.7 ± 8.01 s/clue, expert: 3.1 ± 1.3 s). This finding will be used to estimate memory access time of expert and novice in the model simulation section.

**Figure 4:**
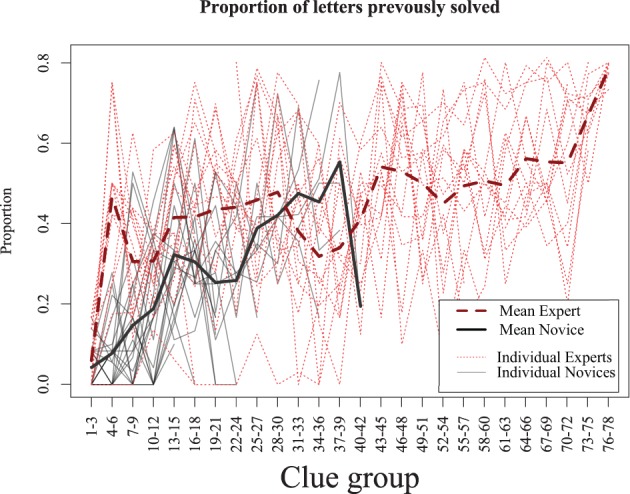
**Proportion of letters previously solved as the puzzle progresses.** Experts (dashed line) solve with 40% partial letters after the first few clues, novice increase slowly and only reach this point when they have completed as much of the puzzle as they are able. Results are averaged across consecutive three-clue blocks.

This shows that experts require less time to solve clues, but does not provide an indication of why. This may be because experts are able to retrieve answers better and more quickly with the same amount of information. To examine this, we computed the proportion of letters completed prior to solving each consecutive solved clue (see Figure 4). For example, if a 6-letter word were solved with three letters that had previously been solved via crossing words, it would be given a score of 0.5, as would an 8-letter word with four letters present. We found that for both experts and novices, as the puzzle progressed, the proportion of previously-answered letters increases. Yet this proportion rose quickly in the experts to around 40% of the word, whereas the novices reached that point only when they had completed nearly all the answers they were capable of. Thus, although the experts may be able to solve clues with a fewer letter hints, they tended not to do so, presumably because solving clues with more letters makes the puzzle easier and solution times faster. We will investigate the implications of this strategy in our model simulations, which we turn to next.

## 5. Model simulation

The basic behavioral results show that experts are much better and faster than novices at lexical and memory access for crossword-related information. They also show that some solution strategies of experts appear to differ from novices. These factors undoubtedly work together to help experts produce superior performance, but it is difficult to cleanly separate them in a naturalistic data set. Consequently, we used the model described earlier to explore the hypothesized differences between experts and novices.

We tested eight distinct models, factorially manipulating strategy (random and optimizing strategies), fluency (two levels of the recovery parameter) and memory access speed (fast and slow), each in order to explain the expert-novice differences. These models are shown in Table 1, in which the smoothing parameters (orthographic σ and semantic σ), likelihood threshold (λ) and search set used values are identical to those determined by Mueller and Thanasuan (2013). We evaluated these models for both competency (ability to solve the puzzle) and resemblance to human data (ability to reproduce effects related to lexical variables and expertise; see Mueller et al., 2007). For these models, two recovery and retrieval time parameter sets were selected as high and low comparisons, and the parameter values were free parameters selected so that they accounted for either expert or novice performance. However, no other deliberate parameter-fitting was conducted, and all other parameters were fixed.

**Table 1:** **Parameters of simulation models**.

**Parameter**	**Models**							
**Code**	**1 OFF**	**2 OFS**	**3 ODF**	**4 ODS**	**5 RFF**	**6 RDF**	**7 RFS**	**8 RDS**
Strategy^1^	O	O	O	O	R	R	R	R
Recovery^2^	15	15	0.5	0.5	15	15	0.5	0.5
Retrieval (s)	0.25	3.0	0.25	3.0	0.25	3.0	0.25	3.0
Reading (s)	1.0							
Typing (s)	0.28							
Moving (s)	0.14							
Smoothing Orth. σ	0.001							
Smoothing Sem. σ	0.00000001							
Likelihood λ	100							
Search set	10							

Three-letter code indicates Strategy (***O***ptimizing or ***R***andom), Recovery (***F***luent or ***D***isfluent), and speed (***F***ast or ***S***low).

1 R, Random movement strategy; O, Optimizing movement strategy.

2 Recovery impacts only the probability of semantic recovery.

### 5.1. Simulated solution strategies

Two solution strategies introduced earlier were examined. The **Random movement strategy** was based on our observation that novice players appeared hunt for clues that were easy to solve, and so their solving strategy appeared haphazard and somewhat random. Although there may have been some nuances not captured by this strategy (e.g., preferring short words; picking clues with fill-in-the-blank patterns), the random strategy picked the next clue at random from the remaining unsolved clues, moved to it, and attempted to solve it. In contrast, we observed that experts tended to make shorter, more deliberate moves from clue to clue, and appeared to solve clues that (1) were close to the current location in the puzzle, and (2) were already partially solved. We adapted a neurocomputational model of search goal selection (Mueller et al., 2013; Perelman and Mueller, 2013) to guide this **Optimizing movement strategy** model. The model first computes weights of each unsolved clue by using Equation (7). The constraints include a cost, which is a distance between the current position and an unsolved clue, and a reward, which is a number of filled letters of each unsolved clue. Then, the strategy decides on the next clue to solve by choosing the largest weighted probability (*Pr_i_*) via a Luce choice rule from Equation (8), where the weights are the estimated discounted proportion of the clue that has already been solved:

(7) $$w_{i}\;=\;{\left(1-\alpha \right)}^{d_{i}}\;*\;(wf_{i}/att_{i}\;+\;s_{1})\;+\;s_{2}$$

(8) $$Pr_{i}\;=\;(w_{i})/\sum_{i\;=\;1}^{x}\left(w_{i}\right).$$

Here, *w_i_* is the weight of unsolved clue *i*; α is a discounting parameter (set to 0.02) indicating how much a potential reward is discounted for each move that must be made; *d_i_* is the distance between the current position to the first position of unsolved clue *i*; *wf_i_* is a number of filled letters of unsolved clue *i*; *att_i_* is a number of times that a model tries to solve clue *i*, *s*_1_ and *s*_2_ are smoothing parameters (set to 0.001 and 0.00001, respectively), that ensure all clues have a non-zero chance of being chosen, and *x* in Equation (8) is the total number of unsolved clues. The basic insight for this calculation is that potential reward, indexed by the ease with which clues can be solved, is discounted via a decaying spreading activation to provide cues about which candidate is the “best closest” clue to attempt.

**Figure 5:**
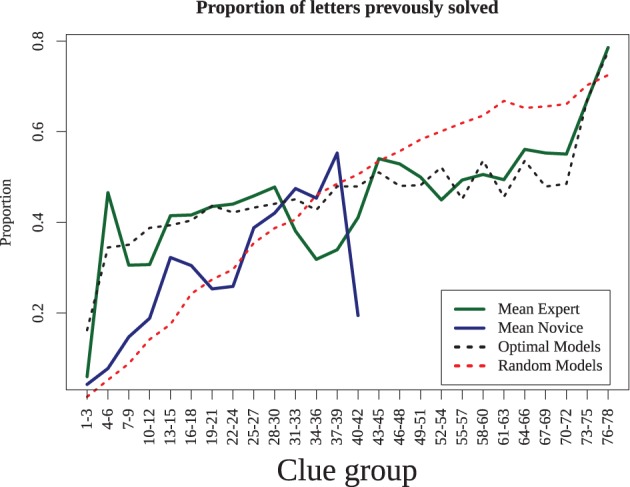
**Mean proportion of letters previously solved for human data (experts and novices) and the simulation results (average of all random models and all optimizing models)**.

By comparing the eight different models, we are able to understand the extent to which different processes may underly superior performance in crossword play. First, to examine the impact of strategy, we analyzed how the proportion of letters previously solved changed as the puzzle was solved, for both human and simulated players (Figure 5). This shows the extent to which players choose (and are able to) solve clues that are already partially completed. Here, the two strategies produce distinct differences that mirrored expert and novice players: the optimizing strategy tended to use more letters almost immediately, whereas the random strategy increased slowly as play progresses.

**Figure 6:**
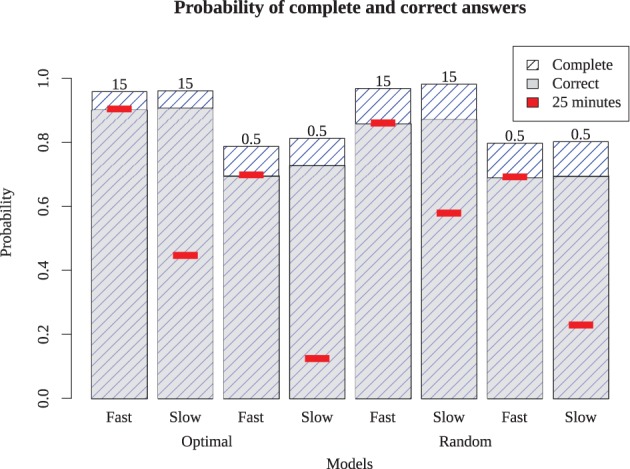
**Proportion of puzzle words completed (highest bars) and completed correctly (gray bars) for the eight different models.** Red inset bars show performance after 25 simulated minutes, indicating that the slow models are able to perform as well as the fast models if given enough time.

**Figure 7:**
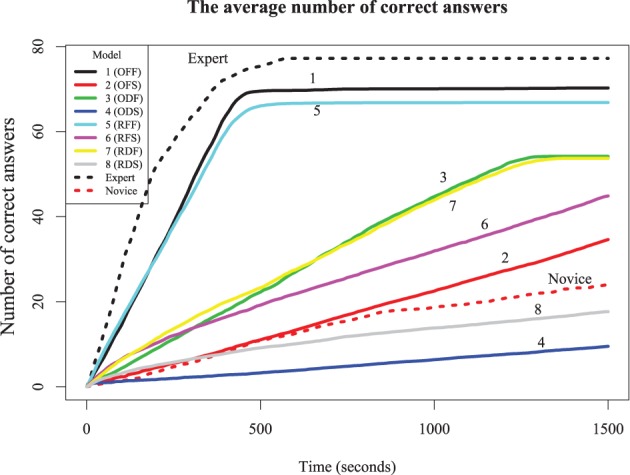
**The average number of correct answers (out of 78) over time for eight models.** Models 1–4 use the optimizing strategy; 5–8 use the random strategy. Odd-number models are fast solution times and even-numbered models are slow solution times. 1,2,5,6 have high fluency, whereas 3,4,7 and 8 have low fluency.

The simulation results in Figure 6 show the probability of complete and correct answers of each model and Figure 7 shows how the mean percentage of the puzzle solved grows over time, for both human and simulated players. Comparing the models to the expert players, only Models 1 and 5 completed the puzzle with timing and accuracy trajectory similar to experts. These models outperformed all human novices, although they did not quite reach the accuracy of experts. These two models have high recovery parameters and fast retrieval times, and differ only in their strategy. Here, the optimizing model is slightly (but not overwhelmingly) better than the random model, suggesting that experts require both fast and fluent retrieval, but their strategy choice may only impact them marginally.

The other six models account for novice play with different combinations of parameters. Models 3 and 7, which have low recovery parameters but fast retrieval times, performed about twice as good as the average novice, and were also better than Models 2 and 6 (which have high recovery parameters but slow retrieval times). This suggests that, at least for our models, overall performance is more sensitive to speed than retrieval fluency. However, Models 3 and 7 asymptote with around 75% of the puzzle complete; wherease Models 2 and 6 will continue to steadily solve the puzzle, and ultimately will complete the puzzle with the same accuracy as the best models, if given enough time. Models that were slow and disfluent (4 and 8) performed worse even most novices, suggesting these provide a lower bound for reasonable performance. The best account of novice players is that they are somewhat slower and substantially less able to retrieve correct responses than experts. Curiously, although the optimizing strategy made only a small difference for the high-fluency models (i.e., those with high recovery parameters), it was paradoxically *worse* than the random strategy for the low-fluency (novice) models. For the non-expert models (and humans), advanced strategies dictating how to solve the puzzle require the solver to have a choice in their solution path. Novices may not really have much of a choice; there may be only a few clues they can easily solve, and so a more exploratory (i.e., random) model may find these sooner than a more deliberate strategy.

**Figure 8:**
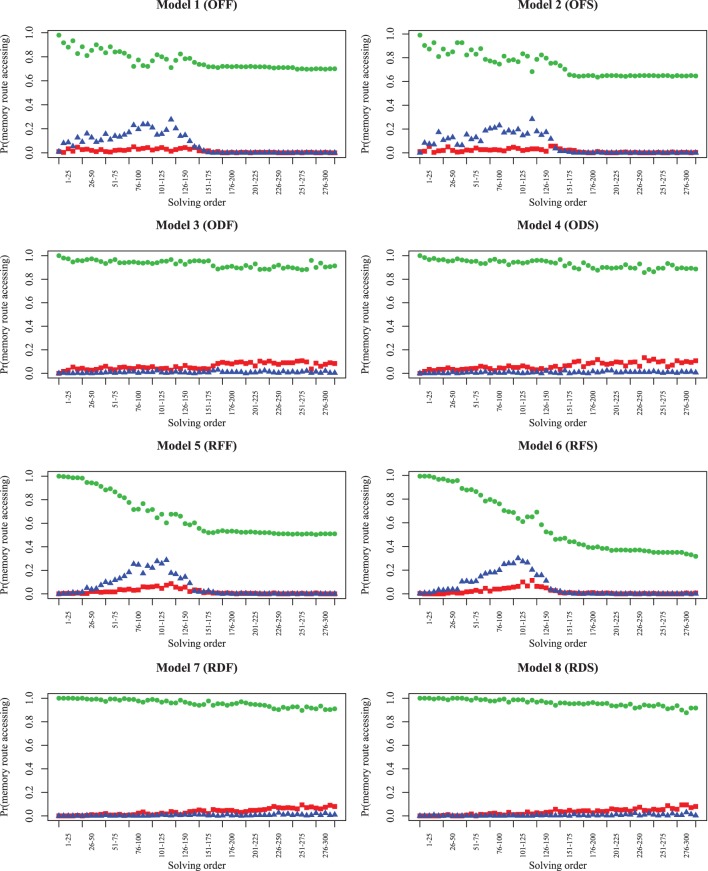
**Model simulations showing the probability of each memory route (or both routes) producing the selected answer (semantic route = green circles; orthographic route = red squares; both = blue triangles).** At each timepoint, the three values sum to 1.0. The trials categorized as “both” indicate that both routes selected the same answer; trials categorized as either orthographic or semantic were ones in which that route alone produced the better answer.

Up until this point, we have primarily examined the probability of completing clues and the entire puzzle over time. To solve each clue, the model uses both orthographic and semantic information. The results indicate that experts adopt strategies that enable them to solve clues with *more* partial letters than novices, and models with poor semantic fluency perform worse when they adopt this strategy. To understand the extent each of these two types of information lead to chosen answer for different models, we examined 100 simulation traces for each model, across 300 consecutive solution attempts as the puzzle was solved. In these cases, we identified the answers produced by orthographic and semantic routes in isolation, to determine the probability of the response arising from each route (Figure 8). Here, because no time limit was imposed, each row of models (differing only in timing) are essentially identical (Model 1/2, Model 3/4, Model 5/6, and Model 7/8). All models show that the semantic route is more likely to produce a preferred answer, indicating that being able to fluently retrieve answers to clues is of primary importance. However, the number of answers that match on the orthographic (red squares) or both routes (blue triangles) increases to around 30% in Model 1/5 and 2/6, and then falls off as the puzzle is completed. The optimizing strategies (Model 1/2) produce this rise earlier in the puzzle, which is consistent with the patterns shown in Figure 5. Even though the recovery parameter only directly impacts only the semantic route, the high-fluency models are able to make orthographic-route solutions possible earlier, and strategies can make these accessible even earlier. We hypothesize that orthographic-route solutions are faster and more automatic as they rely on visual pattern completion. Better semantic skills, coupled with appropriate strategies, help transform the puzzle into one where orthographic cues are more useful.

**Figure 9:**
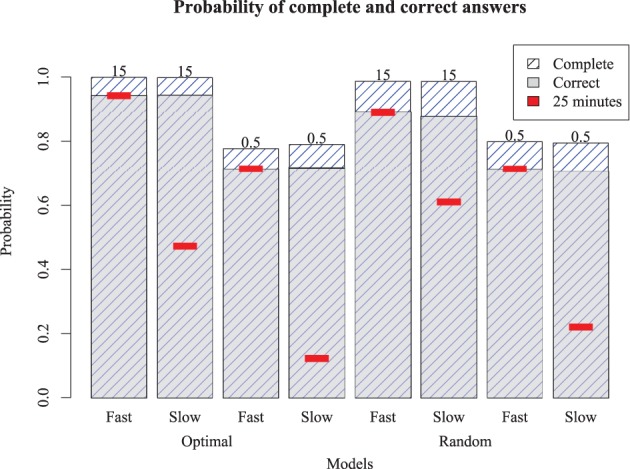
**Model performance on an easy (Monday) puzzle.** Bars show proportion completed and correct for eight different models, with red inset bar showing performance after 25 simulated minutes.

**Figure 10:**
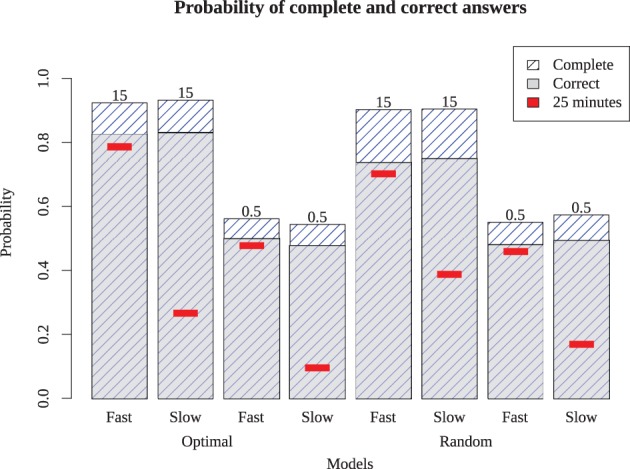
**Model performance on a difficult (Thursday) puzzle.** Bars show proportion completed and correct for eight different models, with red inset bar showing performance after 25 simulated minutes.

To determine whether these results hold more generally, we also ran the models on two additional puzzles: a simple Monday puzzle (February 27, 2012, by Bill Thompson) and a more difficult Thursday puzzle (March 1, 2012, by Steven E. Atwood) published by the New York Times. The results are shown in Figure 9 for the Monday puzzle, and in Figure 10 for the Thursday puzzle. For the Monday puzzle, absolute performance and performance across models is nearly identical to the puzzle tested in our experiment. For the Thursday puzzle, accuracy gets moderately worse, as would be expected because of its greater difficulty. Models 1 and 5 were able to solve these puzzles better than the others, and replicated the finding that the optimizing strategy only improves play for the best models.

## 6. Discussion

The model and experiment we presented here examine what enables humans, and experts in particular, to solve crossword puzzles. Because crossword play is fairly complex, a number of sources could contribute to expert-novice differences. Table 2 highlights these factors, with an assessment of their importance in crossword play. Although we have drawn a number of conclusions from these models, they suggest that differences in semantic knowledge are sufficient to explain expert-novice differences. This includes both the richness of relevant semantic associations, and the ability to fluently retrieve the correct response via these associations. Other factors (including strategy and speed) may differ between experts and novices, but these factors are ineffective or counterproductive without substantial knowledge of the crossword lexicon. Finally, although experts might have better orthographic fluency, this alone cannot explain their superior performance because they actually tend to solve clues with *more* partial letter information than novices. This suggests they may prefer to use orthographic information to solve clues when able, and our analyses indicates that improved semantic fluency actually enables them to do so. Next, we will discuss each of the cognitive factors contributing to crossword solving in greater detail.

**Table 2:** **Cognitive factors that are involved in crossword play, and our evaluation of their relative importance in explaining crossword expertise**.

**Factor**	**Expert-novice importance**	**Comments**
Semantic knowledge	High	Semantic knowledge is the primary avenue to solving crossword puzzles.
Semantic retrieval fluency	High	Solvers need not only to possess the knowledge, but must be able to retrieve and produce it.
Orthographic pattern matching	Small	Having more orthographic information reduces the semantic difficulty, and experts appear to use this strategically.
Error detection	Minor	Models with no error detection can perform close to expert-level and better than novices.
Error correction	Minor	Expert models perform slightly worse than human experts; error correction could reduce this gap.
Backtracking	Minimal	Unlike classic AI, deep backtracking is of little importance for experts.
Solution strategies	Moderate	Strategies help experts solve puzzles more often via orthographic route; these same strategies harm performance in novice models.

**Table 3:** **Clues from puzzle, matching answers in Figure 3**.

• 1A Electrical Rock Band Name	• 72A Walk or trot or gallop, for a hors
• 5A D.C. interest groups	• 73A Monopoly payment
• 9A Space__(airhead)	• 1D Nile serpants
• 14A Make like a tree and leave!	• 2D Spiced tea beverage
• 15A From a different perspective, in a chatroom	• 3D Voodoo __
• 16A Tropical Island neighbor of Bonaire	• 4D Seinfeld's field
• 17A Tree that grows dates	• 5D Many a university lab employee
• 18A Arty spot in lower Manhattan near Tribeca	• 6D Building block of matter
• 19A Ways to send a office messages before e-mail	• 7D Regis and Kelly, e.g.,
• 20A Talking picture's predecessor	• 8D Powerful push
• 22D Abbr. at the end of many company names	• 9D Regained consciousness
• 23A Nonvegetarian sandwich, for short	• 10D Is, for many
• 24A Pet Bo or Barney of the White House	• 11D Food-service elevator
• 25A Dismissed	• 12D Virus named for an African river
• 27A Child on a military base	• 13D Delectable
• 31A Generic vending machine drink choice	• 21D A Duke or Earl
• 32A Letters from one who's low on cash?	• 26D Swiss mountain range peak
• 33A Cowboy cattle catcher	• 27D __ it the truth!
• 35A __ of the litter	• 28D Libertine
• 38A Trail mix ingredient	• 29D It may be pressed for privacy during a conference call
• 39A Massachusetts summer time zone (abbr.)	• 30D Fictional terrier
• 40A Train boarding location (abbr.)	• 34D Department of Labor arm protecting worker safety
• 43A Mai __ (tropical drink)	• 36D It's pushed in a grocery store
• 44A Be abundant with	• 37D Peck on the cheek
• 46A Playwright Fugard	• 41D Young scientist of old teen fiction
• 48A Hospital locations open for treatment at all hours (abbr.)	• 42D Dominant dog
• 49A Dull, dull, dull	• 45D Dodgers' org.
• 52A O'er the __ we watched, were so gallantly streaming	• 47D Cheerful refrain in song
• 54A Fizzy liquid ingredient of many cocktails	• 50D Hockey statistic
• 57A Moo __ pork	• 51D __ cross buns
• 58A Mare's morsel	• 53D Writer
• 59A They may run deep	• 54D Follow-the-leader party dance
• 64A Capone henchman Frank	• 55D Hideouts
• 66A Long length of fashion	• 56D Joltin' Joe
• 67A Informal greeting	• 60D Sixty-two, in roman Numerals
• 68A Increases	• 61D Ireland, in Irish
• 69A First Arabic letter	• 62D Saving Private __
• 70A Kind of thermometer or hygiene	• 63D It comes from a shaker
• 71A Like __	• 65D Only even prime number

### 6.1. Semantic knowledge

Our results suggest that the primary factor separating experts and novices is in their ability to fluently and quickly access memory via semantic cues. The four fluent models (1, 2, 5, and 6) were all able to solve 70–90% of each of the clues from the puzzles we examined (if given enough time). This is consistent with Hambrick et al. (1999), who showed that general knowledge is correlated with crossword solution performance. Logically, this makes sense because orthographic-based cuing is only feasible if enough constraining orthographic information is present, and this is only possible by solving at least some clues using a primarily semantic route.

A number of open questions remain about the access and representation of semantic knowledge in crossword players. Certainly, experts learn information specific to the relationship between clues and responses, and this is exactly the knowledge that our models possess. However, much of this knowledge is general information, some of which consists of general knowledge and trivia (especially person and place names in history, geography, entertainment, pop culture, etc.), and general word meanings. It remains an open question whether experts simply know the crossword-related information better, or whether they possess something else, such as the ability to encode or retrieve general associations, that may benefit them more generally. Anecdotal evidence suggests that experts may be especially good at encoding knowledge or retrieving knowledge learned only once, because many of the top players became great either at a young age, or relatively soon after starting to play seriously.

Although it did not perform as good as the top players, our model does perform better than novice and casual players. This suggests that its knowledge base is probably too rich, or at least too specific to crossword information. Incorporating more non-crossword information would likely make the model worse, as other associations irrelevant to crossword play would compete for retrieval. We have explored incorporating other more general knowledge information, reducing the use of a crossword-specific corpora, but these experiments go far beyond the scope of the research reported here.

### 6.2. Orthographic knowledge

Because experts solve puzzles so quickly, it is tempting to assume that they are relying heavily on visual pattern recognition to fill in possible answers. The problem of this assumption is that some partial information is necessary to solve via an orthographic route, and a puzzle cannot provide these constraints without first solving some clues semantically.

Our analysis suggests that experts play strategically in such a way that increases their chance of using orthographic information, solving words that have at around 40% of the letters complete. This indicates an important role for orthographic information. Other findings (Mueller and Thanasuan, 2013) suggests that experts can use orthographic information, such that if there are three or fewer missing letters, the correct solution can be guessed with above 80% accuracy (even for difficult clues), whereas novices achieve 40-50% accuracy on the same clues. An important consequence of this is that solutions via orthographic information reduce the impact of clue difficulty, and so strategies that encourage orthographic solutions can essentially make a difficult puzzle easier.

### 6.3. Speed

The overall speed with which a player can type, move, and generate responses can explain some of the differences between novice and expert players. Our models attribute all differences to memory retrieval, The slow fluent models (Model 2 and 6) complete the puzzle as well as the fast models if given enough time, but are simply slower. Among experts, the best are both fast and accurate, but as players age they may tend to slow down while remaining accurate. There may also be other aspects of preparation, practice, experience, and genetics that lead speed and accuracy to be dissociable in crossword play.

To examine this more, we looked at the scores of the 2013 American crossword puzzle tournament^4^, which recorded solution times for 572 competitors on 7 puzzles. If we consider only the 2935 (out of 4004) puzzles that were completed within the time limit, the correlation between number of missing letters and time remaining after solving was only −0.12 (indicating that slower solvers tended to make slightly more errors). Although this is statistically significant [*t*(2899) = −6.5, *p* < 0.001], this suggests that the very large difference in solution times are not reflected strongly in errors committed (including all 4004 puzzles raises the correlation to −0.51; this correlation must be stronger because those who did not finish in the allotted time almost always made errors). Consequently, this suggests that there are substantial aspects of speed that are independent of memory retrieval fluency, and it is reasonable to model these as independent sources of expertise.

### 6.4. Gridfill strategy

Although the optimizing strategy we examined was measureably different from the random strategy, its use amounted to small improvement for the fluent model, and actually harmed the novice model. The choice of a solution strategy may shave off precious seconds for an elite solver, but changing one's solution strategy will not generally enable a novice to improve substantially (and may be counterproductive). Furthermore, the strategies experts engage in may not realistically be available to novices; improving speed by deciding how to solve will only work if the player really has a number of options to solve. Novices may not have many true options–there may only be a few clues they can answer at any given time, and so their best strategy is one that attempts to find those earlier.

Our results also suggest that experts' strategies may tend to shift solutions from a semantic-route solution strategy to one that enables the use of orthographic information. Our previous research showed that orthographic solutions can reduce and nearly eliminate the difficulty of the clue, and so to the extent that experts use an “optimizing” strategy, it appears to help increase the chances of an orthographic-route solution that makes difficult clues easy.

### 6.5. Comparison to traditional AI approaches

The most successful AI crossword solvers have worked in ways that are fundamentally different from human solvers. For example, Dr. Fill's strategies are heavily based on constraint satisfaction, and use orthographic and crossing words extensively to constrain possible results. In addition, it takes advantage of the computer's speed, searching through the solution space to solve a puzzle many times before identifying the best solution.

Our present model is not as good at solving as Dr. Fill. Whereas our model solves 80-90% of puzzle clues, Dr. Fill has no problem completing almost any straightforward puzzle. However, our expert model still outperforms average and novice players, and produces performance akin to very good players. The reasons for these differences are instructive, highlighting the additional skills that humans have, and also indicating the extent to which they are important. First, our model does not incorporate any complex rules for tricky theme puzzles (often involving letter substitution, puns, rebuses, and other wordplay). Such rules might be the aspect of Dr. Fill's intelligence that is most human, because they are learned conventions that an expert solver must use to transform the best answer into one that fits the grid. These rules are things that experts learn and use, but they are also things that give novices the most trouble. Yet many puzzles don't even include such tricks, and so although implementing them might be informative about the types of logical processes expert crossword solvers engage in, they may not translate as easily to other domains as does our basic memory access model. Second, the model does not detect or correct errors. It is interesting that our model's performance can nevertheless be very good (and much better than typical novices), even while making errors that prevent later responses from being correct. Third (and related), the model does not perform backtracking. Consequently, it can sometimes get stuck on an incorrect solution that prevents it from completing several other clues in a puzzle, even though the error may be relatively easy to detect. This is informative because traditional AI algorithms using search will typically compensate for uncertainty in generating the correct partial solution with extensive trial-and-error. Experts may only need to do this on occasion, because they are almost always certain of being correct when they make a response.

Overall, although traditional AI solutions to crossword puzzles are both useful for testing AI algorithms, and are a substantial engineering feat, the processes they typically use differ substantially from how experts approach and solve puzzles. In contrast, our model succeeds by using strategies akin to human players; iteratively solving a puzzle, clue by clue, one time. To do so, rather than attempting to make many guesses and letting the web of constraints identify an optimal solution, a decision must be made regarding whether the candidate answer is good in on its own right. This is the essence of recognitional-decision making in many domains, especially for domains requiring exact solutions.

### 6.6. Crossword play and recognitional decision making

The present model shows that the traditional AI approach fails as a reasonable model of human crossword expertise. In contrast, a memory-based retrieval process is used. Such recognitional decision processes are common to many fields of expertise, but the domain of crossword play involves some caveats to earlier models.

First, the core of the RPD model common in the Naturalistic Decision Making community is that cues in the world activate a past workable solution, which may be adapted (via mental simulation) to provide the best course of action. For many expert domains, such solutions are not only common, they may be the only way to proceed. For example, every house fire differs, and so a decision about how to fight the fire based on a past solution must be adapted at least minimally to suit the current situation. Furthermore, there are likely to be dozens of essentially equivalent workable approaches that could be used successfully. In contrast, crossword puzzles only permit a single solution, and so the approach must be different. To some extent, a clue may activate a similar word-clue from the past, or may activate an incorrect answer that is nevertheless semantically similar to the correct one. However, the critical process is one where a generated answer is evaluated for acceptability, and discarded if it won't work, either to continue search on the present problem or to move to a new problem until more information is gained.

This suggests a class of problems for which the classic RPD model must be amended: expert domains requiring or encouraging exact solutions. Although many types of puzzles are examples of these, other domains may involve costs and logistics that make approximate solutions inadmissible or inappropriate. In such cases, the decision space may not permit adapting a candidate decision to fit the current situation, and is likely to involve (1) determining if the current solution is good enough, and (2) continuing to search if not. There are aspects of medical diagnosis and general troubleshooting (e.g., both mechanical and software) that are likely to fit this kind of decision style. In some of these cases, approximate solutions may be ill-advised or untenable, and a decision cannot be made until the exact source of a the problem is identified. Similarly, other domains of expertise afford little opportunity to adapt plans. For example, Veinott and Mueller (2011) examined decision times in NFL quarterbacks, who must sequentially evaluate and discard high-probability low-gain options in favor of later high-gain lower-probability options that are yet to emerge. These favor a decision style in which candidates are retrieved and rejected until an appropriate path is found, and so is conceptually similar to the search problem delineated here.

In conclusion, we have examined expert and novice performance in crossword play, and used a biologically-inspired AI model to understand how some of the underlying processes contribute to crossword play in general, and crossword expertise in particular. Results indicate the importance of semantic retrieval fluency, and suggest that such fluency may be a common property of many knowledge-based expert domains.

## Author contributions

Kejkaew Thanasuan performed primary experiment design and implementation, data analysis, model implementation, and manuscript preparation. STM contributed to experimental design, data analysis, model conceptualization, and manuscript preparation.

### Conflict of interest statement

The authors declare that the research was conducted in the absence of any commercial or financial relationships that could be construed as a potential conflict of interest.

## Footnotes

^1^To be fair, there are classes of clues that are deliberately ambiguous so that the solver is likely to know that one of a small number of responses is correct, but not which one. For example, “Morning hour” ‘- - - A M’ is likely to be ONEAM, TWOAM, SIXAM or TENAM; “Late Month” ‘- - - - M B E R’ could be NOVEMBER or DECEMBER, etc.

^2^For example, if a clue were “Spacey costar” (with the correct answer BYRNE), someone may 1. fail to retrieve the answer even while picturing Gabriel Byrne's face, but also 2. think about the film *The Usual Suspects*, and fail to remember Byrne was in it, even if they would be able to generate the name. In both cases, we presume that the solver would recognize the clue as correct if it were present, but cannot actually generate it.

^3^Other advanced strategies reported by competetive players and observed in our studies include ploys like waiting to correct an error until the end of a puzzle, or deferring entering a solved word to fill it in with the crossing words.

^4^http://www.crosswordtournament.com/2013/standings/tabdelim2.txt
